# Effect of HIF-1α on biological activation of human tongue squamous cell carcinoma SCC-15 cells *in vitro*

**DOI:** 10.3892/ijo.2015.2934

**Published:** 2015-03-26

**Authors:** XIAOKANG ZHOU, DANQING HUANG, ZHONGXIU XUE, XIUHUI XU, KAI WANG, YAO SUN, FEIWU KANG

**Affiliations:** Laboratory of Oral Biomedical Science and Translational Medicine, School of Stomatology, Tongji University, Middle Yanchang Road 399, Shanghai 200072, P.R. China

**Keywords:** hypoxia-inducible factor-1α, tongue squamous cell carcinoma, deferoxamine mesylate, RNA interference, lentiviral vector

## Abstract

Hypoxia-inducible factor-1α (HIF-1α) is a key regulator for tumor cells and tissues to adapt to hypoxic condition. Suppressing the expression of HIF-1α is important to evaluate its effect on cancer cells. This study was carried out to analyze the effect of HIF-1α on the biological activation of human tongue squamous cell carcinoma (TSCC) SCC-15 cells. In this experiment, deferoxamine mesylate (DFO) was used to induce hypoxic condition. HIF-1α gene was suppressed by lentiviral vector. The effect of the level of HIF-1α expression was tested on the proliferation, cell cycle, cell apoptosis and cell invasion of SCC-15 cells. We demonstrated that SCC-15 cells showed a more aggressive phenotype after treated with DFO. Additionally, DFO was able to induce the expression of HIF-1α protein. Lentiviral vector can effectively inhibit HIF-1α expression on mRNA and protein level. Under normoxic or hypoxic conditions, downregulation of HIF-1α for SCC-15 cells induced cell apoptosis and inhibited growth and invasion. These results showed that suppressing the expression of HIF-1α inhibited the aggressive potential of SCC-15 cells under normoxic and hypoxic condition. Thus, finding an effective and safe pathway to inhibit the expression of HIF-1α can help us to improve the survival rate of human TSCC patients.

## Introduction

Cells with indefinite proliferation, spreading to adjacent tissues, regional lymph nodes and distant organs are characteristics of cancer. Among the oral and maxillofacial cancers, squamous cell carcinoma is the most common one. Every year >410,000 new oral squamous cell carcinoma patients are diagnosed, accounting for 1–5% of all cancers (1). In oral malignant tumors tongue squamous cell carcinoma (TSCC) is the most common cause of cancer-related deaths. Although chemotherapy, radiotherapy, and surgical therapy for TSCC have developed rapidly in the past years, the 5-year survival rate is still poor (2,3). Most cancers including TSCC are considered as a gene-related disease and associated with the activation of oncogenes and inactivation of tumor-suppressor genes. Hence, finding a safe and effective therapy to change the abnormal expression of genes and to improve the rate of survival with TSCC is imperative. RNA interference (RNAi) has emerged as a powerful method for gene suppression in molecular medicine. RNAi is the process of silencing genes by the sequence specific double-stranded RNA (dsRNA). Hence it is post-transcriptional gene silencing in animals and plants. Fire and Mello were awarded the Nobel Prize for Medicine in 2006 for discovering RNAi in 1998 (4). Studies have shown that RNAi is a promising anticancer therapeutic tool (5,6).

The center of the solid tumor is often in a hypoxic microenvironment because of its rapid growth (7). The hypoxic conditions can lead to a more malignant tumor. It can enhance abnormal angiogenesis, invasion, metastasis of tumors, and result in poor prognosis (8,9). To adapt to the hypoxic microenvironment, many normal and abnormal factors are regulated, including hypoxia-inducible factor-1(HIF-1) which plays an important role in the process. HIF-1, a transcription factor was found in 1992 (10). It is composed of two subunits, a strictly regulated α subunit and a constitutive β subunit, HIF-1β is also called aryl hydrocarbon receptor nuclear translocator (ARNT) (11). HIF-1β levels of mRNA and protein are maintained constant regardless of oxygen tension (12), whereas, HIF-1α is an oxygen-liable subunit. In normoxia, HIF-1α can be degraded by rapid ubiquitination [its protein has a short half-life (*t*1/2~5 min) under normoxia (13)]. However, under hypoxic conditions, the decay of HIF-1α is suppressed, and then it can translocate into the nucleus and dimerizes with HIF-1β and forms the active complex HIF-1 (14). The activated complex associate with hypoxia response element (HRE) to induce expression of its target genes (15). The target genes, including erythropoiesis, glycolysis and angiogenesis (16), are essential for tumors to adapt to and survive in hypoxic conditions. Previous studies have found overexpression of HIF-1α in various human cancers may play an important role for cancer progression (17,18), which implied that HIF-1α is an essential transcriptional regulator of tumor microenvironment. Therefore, gene silencing HIF-1α by RNAi may be an effective method to control the malignancy of tumors and improve the survival of patients.

Previously it was found that HIF-1α might be a significant prognostic predictor for TSCC patients (19). Another study showed that HIF-1α can regulate angiogenesis and survival of oral squamous cell carcinoma (20). Also, we that HIF-1α was expressed in oral squamous cell carcinoma, and found that the levels of HIF-1α in human TSCC seemed to be correlated with human prognosis (21). These findings implied that HIF-1α is an important factor in development and treatment of TSCC. In the present study, according to the principles of RNAi, we constructed lentiviral vector targeting HIF-1α and infected TSCC cell line SCC-15 cells to investigate the effect of HIF-1α on the biological behavior of SCC-15 cells.

## Materials and methods

### Cell lines and reagents

Human TSCC SCC-15 cell line was provided by Dr Huang Xin, Beijing Stomatological Hospital, Capital Medical University, Beijing, China. SCC-15 cells were cultured in Dulbecco’s modified Eagle’s medium/F12 (DMEM/F12=1:1). Medium was supplement with 10% fetal bovine serum (FBS), 100 U/ml penicillin, 100 μg/ml streptomycin. The cells were incubated at 37°C in a humidified atmosphere with 5% CO_2_. Hypoxia was induced by 100 μmol/l deferoxamine mesylate (DFO), with 1% O_2_ balanced with N_2_.

### Construction of lentiviral vector mediated RNAi

We use siRNA design software (www.ambion.com) to choose the RNAi target gene sequence. The target gene sequence of HIF-1α (NM_001530) is GATGAAAGAATTACCGAAT. The control target sequence is TTCTCCGAACGTGTCACGT. In this experiment, we generated the double-stranded oligonucleotides targeting the endogenous HIF-1α gene cloned into GV248 vector (hU6-MCS-Ubiquitin-EGFP-IRES-puromycin), and named it lentiviral/shRNA-HIF-1α (LV-shHIF-1α) (Table I). The sequence was not related to HIF-1α sequence which was designed and used as negative control and termed letiviral/shRNA-control (LV-shCon) (Table I). The two vectors were confirmed by DNA sequencing.

### Lentiviral vector production

According to the manufacturer’s instructions, first, the packaging 293T cells were trypsinized, collected, and resuspended at a density of 1.2×10^7^ cells/20 ml in growth medium containing 10% serum. The cells were seeded in a 15-cm dish. Next, DNA-Lipofectamine 2000 complexes containing 20 μg pGC-LV vector, 15 μg pHelper 1.0 vector, 10 μg pHelper 2.0 vectors and 100 μl Lipofectamine 2000 were prepared and mixed with Opti-MEM medium ≤5 ml, and incubated at room temperature for 20 min. After the 293T cells reaching 80% confluence, the medium was replaced with serum-free medium. Two hours later, the DNA-Lipofectamine 2000 complexes were added to the serum-free medium and incubated at 37°C in a humidified atmosphere with 5% CO_2_. Eight hours later, the medium was removed and replaced with the fresh medium containing 10% serum. Forty-eight hours later, the medium containing lentivirus was centrifuged at 4000 g for 10 min at 4°C to pellet cell debris, and to concentrate the lentivirus at 4000 g for 15 min and stored at −80°C.

### Transduction of target cells and selection

SCC-15 cells (2×10^5^) were collected and seeded in each well of a 6-well plate with 1 ml of complete media and transduced by lentiviral vectors at a MOI (Manual Optical Inspector) 10. Transduction was carried out with 5 μg/ml of Polybrene. Twelve hours later the medium was removed and replaced with fresh, complete medium. Seventy-two hours later, the medium was replaced with fresh, complete medium containing 2 μg/ml of puromycin to select for stably transduced cells. The medium was replaced with fresh medium containing puromycin every 3 days until puromycin-resistant colonies were identified. The cells transducted with lentiviral vectors targeting HIF-1α were named as RNAi cells. The LV-shCon affected cells were named as Con cells, and the untransducted cells were named as Mock.

### Real-time RT-PCR analysis for HIF-1α

RNAi, Con and Mock cells were treated with or without DFO for 24 h. Total RNA was extracted by using TRIzol reagent (Invitrogen, USA). Additionally, cDNA was reverse transcripted by Transcriptor First Strand cDNA Synthesis kit (Roche, USA). HIF-1α mRNA expression was evaluated quantitatively by real-time RT-PCR Faststart Essential DNA Green Master kit (Roche) and LightCycler^®^ 96 Instrument system (Roche). The thermocycler conditions were preincubation at 95°C for 60 sec, 45 cycles of 95°C for 10 sec, 60°C for 10 sec and 72°C for 20 sec, melting of 95°C for 10 sec, 65°C for 60 sec and 97°C for 1 sec. Reactions were run in triplicate and repeated three times. As an internal control of each sample, the β-actin gene was used for standardization. The relative mRNA expression level of the gene was calculated using the 2^−ΔΔCt^ method. The primers were synthesized (Invitrogen) and the sequences were as follows: HIF-1α: forward, 5′-GTCGCTTCGGCCAGTGTG-3; reverse, 5′-GGAAAGGCAAGTCCAGAGGTG-3′. β-actin: forward, 5′-TGGCACCCAGCACAATGAA-3; reverse, 5′-CTAAGTCATAGTCCGCCTAGAAGCA-3′.

### Western blot analysis

After the three groups of cells were treated with or without DFO for 24 h. Cells were washed with ice-cold PBS twice, treated with buffer [50 mmol/l Tris-HCl (pH 7.5), 5 mmol/l EDTA, 150 mmol/l NaCl, 0.5% Triton X-100, 10 mmol/l sodium fluoride, 20 mmol/l h-mercaptoethanol, 250 mmol/l sodium orthovanadate, 1 mmol/l phenylmethylsulfonyl fluoride], and incubated at 4°C for 30 min. The lysates were centrifuged at 10,000 g for 10 min, followed by collecting the supernatants and stored at -80°C. Protein concentrations were examined by bicinchoninic acid assay methods (BCA protein assay kit, Thermo, USA). Equivalent amount of protein were loaded into 8% SDS-PAGE gels and electroblotted onto PVDF membrane (Sigma, USA). The PVDF membrane was washed three times with TBST solution (0.1% Tween-20 in TBS, pH 7.5) and blocked it for 1 h with 5% skim milk in TBST at room temperature. Subsequently, the membrane was incubated at 4°C overnight with antibodies against HIF-1α (CST, USA, 1:1,000). Following washing with TBST three times, for 10 min, the membrane was incubated with secondary antibody against rabbit at room temperature for 1 h and washed with TBST three times, 15 min each. Each sample was also probed with an anti-β-actin antibody (CST, 1:1,000) as a loading control. Bands were visualized by SmartChem™ Image Analysis System (Sagecreation, China).

### Cell proliferation assay

The proliferation of the cells was assessed by the CCK-8 (Dojindo, Kumamoto, Japan) assay. The cells were collected and diluted into 50,000 cells/ml, then plated with 100 μl cell suspension in each well (n=5) of 96-multiwell plates (Corning, USA). Cells were treated with or without DFO and then cultured for 24, 48 and 72 h before the addition of 10 μl of CCK-8 to the culture medium in each well. After 1-h incubation at 37°C, the optical density of each well was measured with an infinite M200 reader (Tecan, Austria) at 450 nm. Cell viability = (ODexperiment-ODblank)/(ODcontrol-ODblank). Each experiment was repeated three times.

### Cell apoptosis and cell cycle analysis

Three groups of cells were treated with or without DFO for 24 h. After treatment the cells were trypsinized and washed twice with PBS and resuspended with 500 μl binding buffer. Then 5 μl of Annexin V-KeyFlour647 antibody and 5 μl of 7-ADD was added, and incubated for 15 min at room temperature in the dark according to the manufacturer’s protocol prior to FACS analysis. Both early and late stages of apoptotic cells were detected by a flow cytometer of FACSVerse™ (BD, USA).

Mock, Con and RNAi cells were plated in 6-well plates (1×10^5^/well) after reaching 80% confluence; the cells were treated with or without DFO, and then incubated for 24 h at 37°C in a humidified atmosphere with 5% CO_2_. Cells were washed in ice-cold PBS and harvested by trypsinization. The cells were washed with ice-cold PBS twice and fixed with 75% pre-cold ethanol overnight at 4°C. The cells were centrifuged at 2,000 rpm for 5 min, the supernatant was discarded. One hundred microliters of RNaseA (200 μg/ml) was added to the cells for 30 min at 37°C, then 400 μl propidium iodine (10 μg/ml) was added to the cells for 30 min at 4°C in the dark. Flow cytometry was used for analysis by FACSVerse™ (BD).

### Cell invasion assay

Invasion of the three groups of cells were determined by using transwell chambers (24-well plates, 8-μm pore size, Corning). After reaching 80% confluence, cells (1×10^5^) were trypsinized, resuspended and loaded into the inner chamber containing 200 μl serum-free DMEM/F12 medium with or without 100 μmol/l DFO. The lower chamber was added with DMEM/F12 medium (500 μl) containing 10% FBS. The Matrigel gel (Corning) was diluted by serum-free DMEM/F12 medium and placed on the surface of filtration membrane of the transwell chambers. After 24 h of incubation at 37°C, the non-invading cells and the Matrigel gel in the inner chamber were gently removed by a cotton-tipped swab, and the invasive cells were gently washed by PBS. Then the invasive cells were fixed with methanol for 20 min and stained with 0.1% crystal violet for 15 min. The number of invasive cells was counted with five random fields under a light microscope at ×200 magnification. Results were presented as the mean percentage of the control group. Experiments were done in triplicate and repeated three times.

### Statistical analysis

Statistical analysis was calculated using SPSS 20.0 software. Data are presented as the means ± SD. Student’s t-test was used for statistical analysis, with significant differences determined as p<0.05.

## Results

### Expression of HIF-1α in SCC-15 cells and regulation by DFO

**Figure 1:**
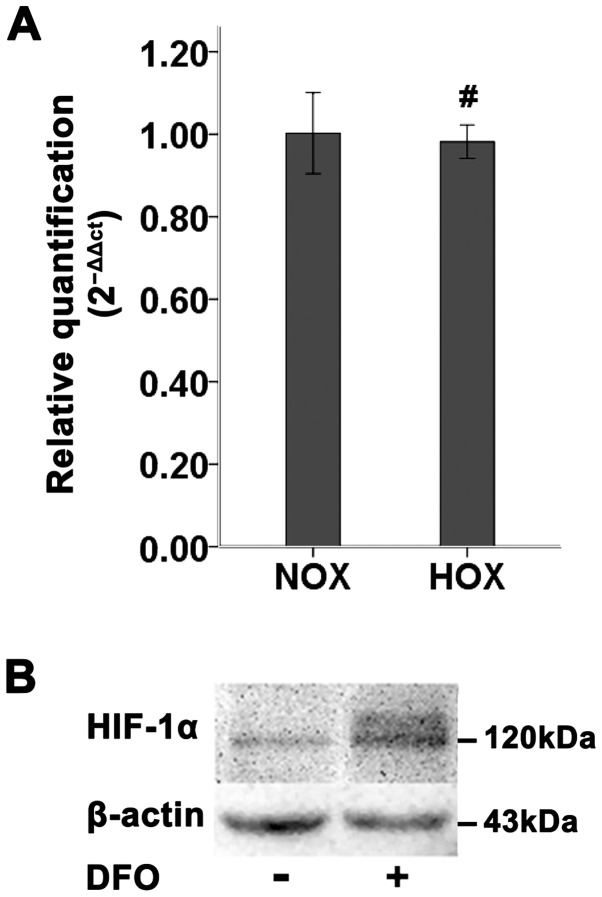
The effect of deferoxamine mesylate (DFO) on the expression of HIF-1α mRNA and protein. (A) The mRNA expression of HIF-1α was analyzed quantitatively by quantitative real-time RT-PCR. SCC-15 cells were treated with or without DFO (100 μmol/l) for 24 h. (B) Whole cell protein was analyzed for HIF-1α protein expression by western blotting. β-actin protein was used as a loading control. SCC-15 cells were treated with or without DFO (100 μmol/l) for 24 h (bars, ± SD, ^#^p>0.05, compared to normoxic Mock group). NOX, normoxia; HOX, hypoxia.

The level of HIF-1α mRNA in SCC-15 cells under normoxic or hypoxic condition was analyzed by real-time RT-PCR. After treated with DFO, no significant change in HIF-1α mRNA transcript was observed (Fig. 1A). However, the level of HIF-1α protein tested by western blotting demonstrated that DFO induced a significant increase of HIF-1α protein in SCC-15 cells ≤24 h (Fig. 1B). The observed molecular weight of HIF-1α protein was detected at 120 kDa (Fig. 1B).

### Lentiviral vector can effectively suppress the expression of HIF-1α

**Figure 2:**
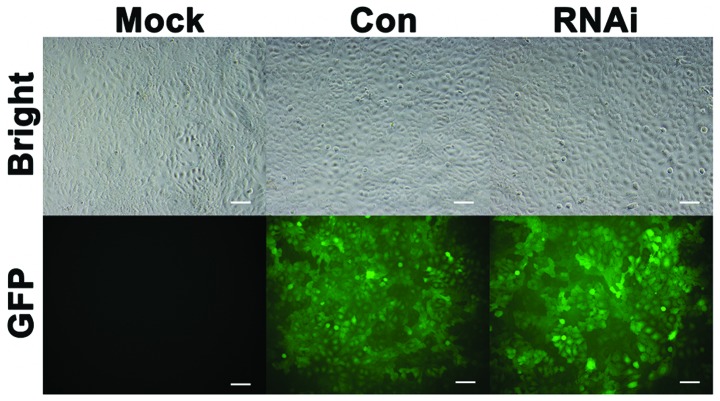
Green fluorescent protein (GFP) expression of SCC-15 cells transduced with lentiviral vector. GFP expression of SCC-15 cells in the Con and RNAi was observed and photographed under an inverted fluorescence microscope. Bar, 100 μm.

**Figure 3:**
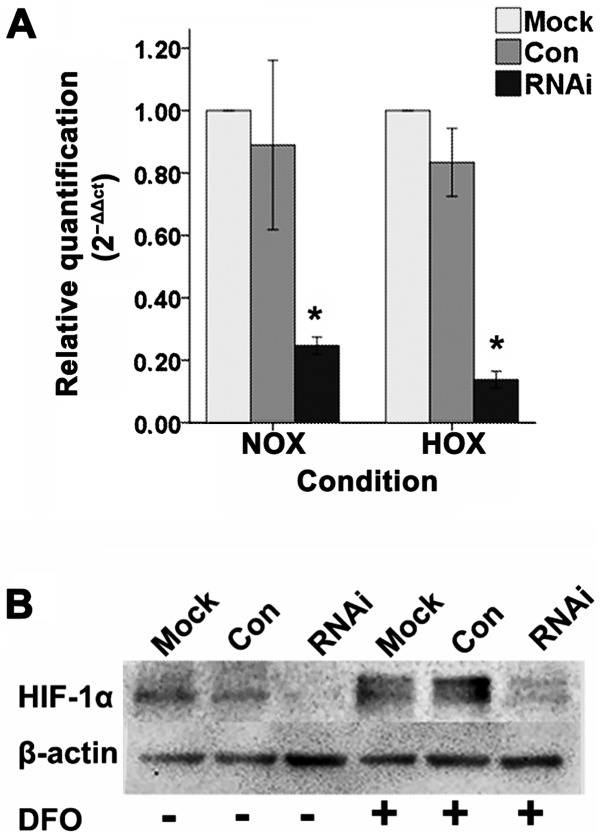
The effect of LV-shHIF-1α treatment on HIF-1α expression in SCC-15 cells. (A) Lentivirus can effectively suppress the expression on the level of HIF-1α mRNA in both normoxia and hypoxia. (B) Western blot result showed that HIF-1α protein was significantly decreased under normoxic and hypoxic condition compared to Mock and Con groups (bars, ± SD, ^*^p<0.05, compared to Mock and Con cells). NOX, normoxia; HOX, hypoxia.

Lentiviral vector affected the SCC-15 cell lines (Fig. 2). Quantitative real-time RT-PCR (Fig. 3A) and western blotting (Fig. 3B) results showed that the mRNA and protein expression levels of HIF-1α in RNAi cells were significantly inhibited compared to Mock and Con cells under normoxic and hypoxic condition *in vitro*.

### Cell proliferation

**Figure 4:**
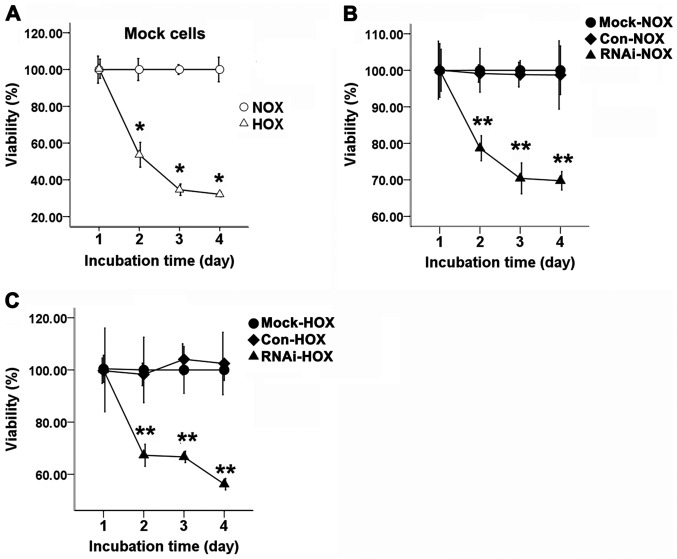
The effect of HIF-1α on cell viability of SCC-15 cells. (A) The viability for Mock cells treated with DFO compared to normoxic cells [Cell viability = (ODHOX-ODblank)/(ODNOX-ODblank)]. (B) The difference of viability between Mock, Con and RNAi cells in normoxia [Cell viability = (ODexperiment-ODblank)/(ODMock-NOX-ODblank)]. (C) The difference of viability between Mock, Con and RNAi cells under hypoxic condition [Cell viability = (ODexperiment-ODblank)/(ODMock-HOX-ODblank)] (bars, ± SD, ^*^p<0.01, compared to normoxic Mock group, ^**^p<0.01, compared to Mock and Con cells). NOX, normoxia; HOX, hypoxia.

The three groups of cells were cultured with or without DFO for 24, 48, or 72 h. When SCC-15 cells were treated with DFO, the proliferation of hypoxic cells was significantly inhibited compared with the normoxic cells (Fig. 4A). The proliferation of RNAi cells was significantly inhibited compared to the Con and Mock cells under normoxic (Fig. 4B) and hypoxic conditions (Fig. 4C).

### Cell cycle

**Figure 5:**
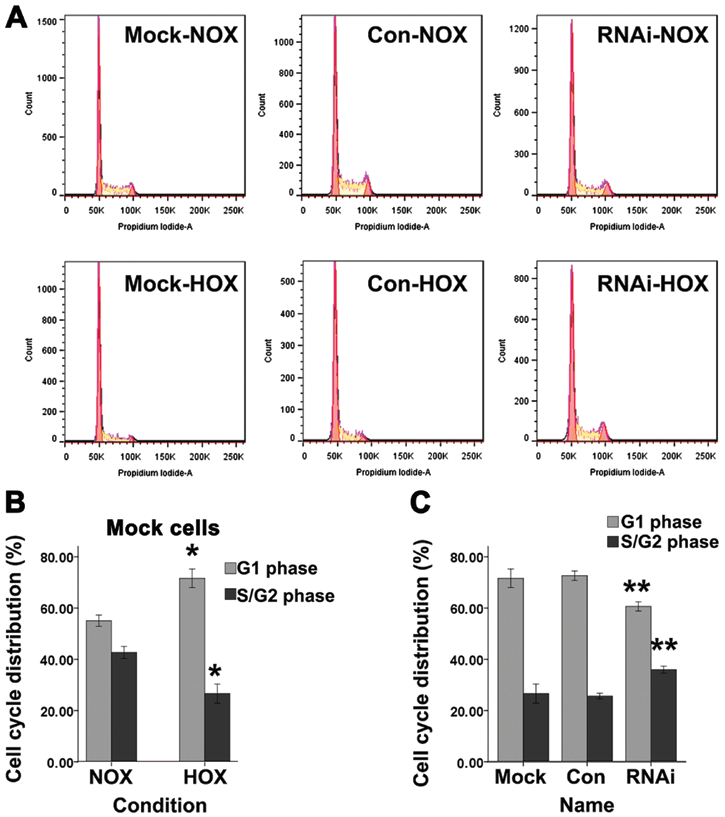
The effect of HIF-1α on cell cycle of SCC-15 cells. (A) Cell cycle of the Mock, Con and RNAi cells were tested by FACS. (B) DFO can significantly increase the number of G1 phase cells and decreased the number of S/G2 phase cells. (C) SCC-15 cells treated with lentiviral vector targeting HIF-1α showed a significant increase on G1 phase cells, and a significant decrease on S/G2 phase cells (bars, ± SD, ^*^p<0.05, compared to normoxic Mock cells, ^**^p<0.05, compared to Mock and Con cells). NOX, normoxia; HOX, hypoxia.

Cell cycle for cells in G1 phase or S/G2 phase was tested by FACS with DNA staining by propidium iodide (Fig. 5A). DFO can induce a significant increase of SCC-15 cells in G1 phase and a decrease of cells in the S/G2 phase compared to normoxic cells (Fig. 5B). SCC-15 cells treated with lentiviral vector targeting HIF-1α showed a significant decrease of G1 phase cells and an increase of S/G2 phase cells compared to Mock and Con cells after treated with DFO for 24 h (Fig. 5C).

### Cell apoptosis

**Figure 6:**
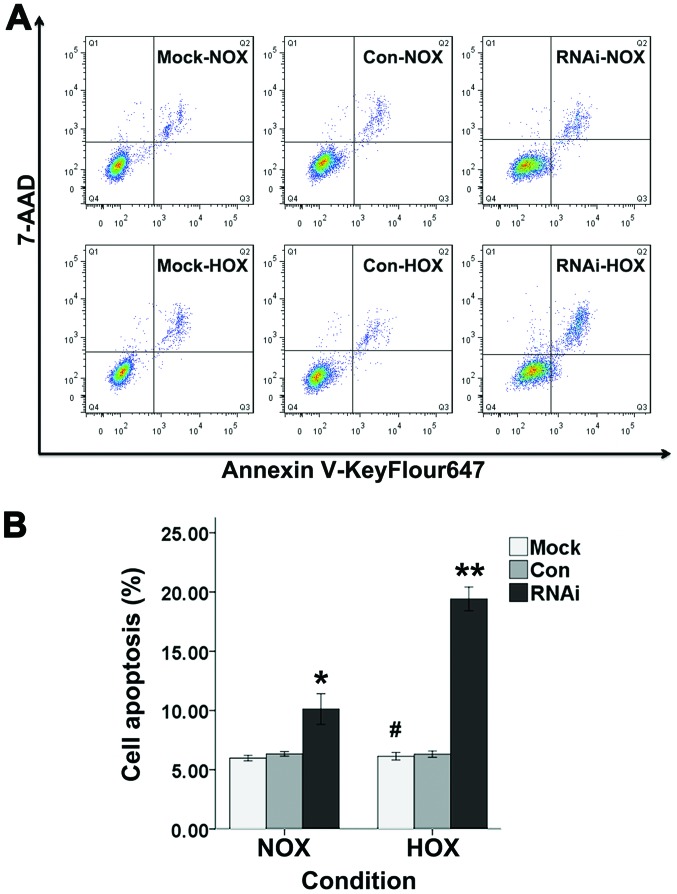
The apoptosis rate of Mock, Con and RNAi cells were evaluated by FACS. (A) The three groups of cells were treated with or without DFO for 24 h. Annexin V-KeyFlour647 and 7-AAD-staining of cells were performed and followed by flow cytometeric analysis. Cells in Q1 quadrant represent cells undergoing necrosis, Q2 quadrant represent late stage of apoptosis, Q3 quadrant represent early stage of apoptosis and Q4 quadrant represent viable cells. (B) The percentage of cell apoptosis for Mock, Con and RNAi cells in normoxia and hypoxia (bars, ± SD, ^#^p>0.05, compared to normoxic Mock cells, ^*^p<0.05, compared to Mock and Con cells in normoxia, ^**^p<0.01, compared to Mock and Con groups in hypoxia). NOX, normoxia; HOX, hypoxia.

Cell apoptosis was evaluated by FACS with Annexin V-keyFlour647 Apoptosis Detection kit (Fig. 6A). There was no significant change in the percentage of apoptotic cells after SCC-15 cells were cultured with DFO for 24 h when compared with non-treated incubated cells (Fig. 6B). It was revealed that the apoptotic cells for RNAi cells were significantly increase compared to Mock and Con cells when incubated with or without DFO for 24 h (Fig. 6B).

### Cell invasion

**Figure 7:**
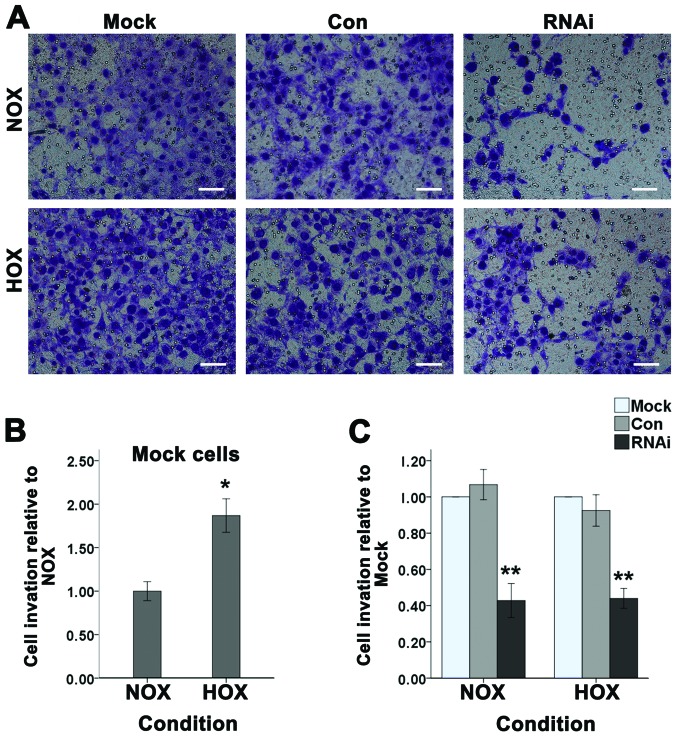
The effect of HIF-1α on cell invasion of SCC-15 cells for 24 h under normoxic or hypoxic condition. (A) The cells passing through the Matrigel basement membrane of transwell chambers were stained by crystal violet, and the staining cells represent the invasion ability of cells (bar, 100 μm). (B) DFO can induce cell invasion of SCC-15 cells. (C) Downregulation of the expression of HIF-1α inhibits the invasion of SCC-15 cells in normoxia and hypoxia (bars, ± SD, ^*^p<0.05, compared to normoxic Mock cells, ^**^p<0.05, compared to Mock and Con groups). NOX, normoxia; HOX, hypoxia.

Studies have shown that HIF-1α can regulate the invasion of tumor cells (22). To investigate the effect of HIF-1α on cancer cell invasion of SCC-15 cells *in vitro*, we used the transwell assay (Fig. 7A). After the three groups of cells were treated with or without DFO and incubated in the transwell chamber for 24 h, 0.1% crystal violet staining was used to detect the number of cells to pass through the Matrigel membrane which represent the invasive abilities. As shown, after treated with DFO, the number of SCC-15 cells passing through the Matrigel membrane significantly increased (Fig. 7B). When HIF-1α was suppressed, the number of cells was significantly decreased compared to the Mock and Con cells under normoxic or hypoxic conditions (Fig. 7C).

## Discussion

Oxygen is one of essential factors for cells and tissues to maintain their function. Hypoxia-inducible factor-1 (HIF-1), a transcription factor, is a key regulator for cells and tissues to adapt to and survive from hypoxia (11,23). HIF-1 contains HIF-1α and HIF-1β, and HIF-1α is highly regulated by oxygen (13). DFO has been proved to induce expression of HIF-1α (24). In the present study, we used DFO to simulate the hypoxic condition, and analyzed the effect of HIF-1α on proliferation, cell cycle, apoptosis and invasion of SCC-15 cells which were correlated to the growth, survival and metastasis of tumors. When the SCC-15 cells were cultured with DFO (100 μmol/l), the expression of HIF-1α was significantly increased, which was similar to our previous study in SCC-6 cells (25). After treated with DFO, the proliferation of SCC-15 cells was inhibited, which was like the other cell types (26). We observed a significant increase of cells in G1 phase and a decrease of cells in the S/G2 phase in DFO-treated SCC-15 cells, which indicated the G1 arrest. However, there was no significant change in the percent of apoptotic cells. These findings were similar to the other cell types in hypoxia (27), which meant a hypoxia-induced growth arrest in SCC-15 cells. The invasion ability of SCC-15 cells was significantly increased after treated with DFO. Therefore, HIF-1α may be an important regulator for the different biological characteristics of SCC-15 cells.

It has been reported that HIF-1α can be regulated by DFO (24). In the present study, we found the level of HIF-1α protein increased after treated with DFO. Then HIF-1α associates with HIF-1β to form HIF-1, and HIF-1 activates hundreds of target genes to regulate angiogenesis, blood vessel tone, vascular remodeling; cell proliferation and viability; erythropoiesis and iron metabolism; glucose transport and glycolysis (11). Therefore, HIF-1α is very important for cells to adapt to hypoxia. Studies found that HIF-1α is overexpressed in most of human tumor cells (18,28). In head and neck carcinomas, study showed that the expression of HIF-1α protein is associated with the microvessel vascular density and vascular endothelial growth factor (VEGF) expression (28). In our previous study, we also found HIF-1α was overexpressed in oral squamous cell carcinoma (21). In the present study, we found that HIF-1α enhanced malignant phenotype of SCC-15 cells.

The hypoxia-inducible transcription factor α subunit was found to be upregulated in DFO-treated SCC-15 cells. We demonstrated that the level of HIF-1α mRNA was not significantly changed between DFO treated and normal SCC-15 cells. However the level of HIF-1α protein was significantly increased after treated with DFO up to 24 h. Study showed that the level of HIF-1α protein is essential for tumor cells to adapt to and survive from hypoxic microenvironment (29). HIF-1α is a key regulator of hypoxic regulation, and it was upregulated mainly on protein level in SCC-15 cells after treated with DFO, indicating that overexpression of HIF-1α was an essential factor for SCC-15 cells to show aggressive phenotype under hypoxic condition. We used RNAi technique to suppress the expression of HIF-1α, and found that silencing HIF-1α can significantly decrease the aggressive potential for SCC-15 cells in hypoxia.

In the present study, we constructed a lentiviral vector to suppress the expression of HIF-1α, which helps to evaluate the function of HIF-1α in progression of SCC-15 cells in hypoxia. SCC-15 cells were transduced with lentiviral vector targeting HIF-1α mRNA. The efficacy of interference was assessed by real-time RT-PCR and western blotting. Results showed that the level of HIF-1α mRNA and protein was significantly suppressed compared to Con and Mock cells in both normaxia and hypoxia.

Although our study showed the association between HIF-1α and proliferation, the mechanism is still not fully understood (30). Hypoxia can arrest tumor cells proliferation (31), however, another study showed that the increased hypoxic stress can accelerate the growth of HIF-1α tumors (27). In the present study, we found a significant increase of the G1 phase cells after treated with DFO. It is suggested that DFO can arrest SCC-15 cells in G1 phase. We used RNAi to inhibit the expression of HIF-1α protein, and test the effect of HIF-1α on the cell cycle of SCC-15 cells. We found a significant decrease of G1 phase cells and increase G2/S phase cells compared to the Mock and Con cells under hypoxia. Hence, HIF-1α was the master regulator for SCC-15 cells to adapt to the hypoxic condition.

Furthermore, hypoxia can not only be associated with cell proliferation but also with cell apoptosis in some circumstances. Hypoxia can induce apoptosis, where HIF-1 plays a complex role (27). It was found that HIF-1α was related to apoptosis (32). It has been reported that severe or prolonged hypoxic condition can maintain the stabilization of p53 protein by HIF-1α stimulation, and leads to induction of apoptosis (33). However, another study showed that HIF-1α might inhibit the hypoxia-induced apoptosis (29). These studies suggest that HIF-1α protein plays a key role in mediating apoptosis of tumor cells. We found that there is no significant change between normal SCC-15 cells and hypoxic cells. RNAi technique was used to evaluate the effect of HIF-1α on apoptosis of cultured human SCC-15 cells under normaxia and hypoxia, and we found the number of apoptotic cells was significantly increased in RNAi cells compared to that of Mock or Con cells under normoxic and hypoxic condition. These results suggested that HIF-1α has an important function in apoptosis of SCC-15 cells.

Hypoxia is a key factor for cancer metastasis (34). Cell invasion is involved in tumor metastasis and progression. In a glioma cell line, gene silencing of HIF-1α can downregulate MMP-2/MMP-9 to suppress cell migration and invasion into adjacent normal tissue (35). In the present study, we analyzed the effect of HIF-1α on cell invasion of SCC-15 cells by a transwell assay *in vitro*. In hypoxia, the number of SCC-15 cells passing through the extracellular matrices (ECM) significantly increases compared with the normoxic cells. Treatment with RNAi lentiviral vector, decreased significantly the amount of cells passing through the Matrigel gel. Thus, HIF-1α may play a major role in regulating the hypoxia-induced invasion of SCC-15 cells to Matrigel *in vitro*.

In conclusion, our results showed that hypoxic SCC-15 cells expressed high level of HIF-1α protein, and were invasive, whereas downregulation of HIF-1α by RNAi technique can decrease the malignancy of SCC-15 cells. Our study suggests that lentiviral vector target HIF-1α specifically suppressed HIF-1α gene expression, induced cell apoptosis, and inhibited the growth and invasion of SCC-15 cells. Hence gene silencing HIF-1α can consequently reduce the malignant potential of SCC-15 cells. Our present study showed the expression of HIF-1α can regulate proliferation, cell cycle, apoptosis and invasion. Thus, the expression of HIF-1α is an essential factor for human TSCC to regulate its malignancy. The RNAi target HIF-1α can be a potential therapeutic measure and effective tool to improve the survival of TSCC patients.

## Figures and Tables

**Table I:** The sequence of double-stranded oligonucleotides for LV-shHIF-1α and LV-shCon.

Name	Sequence
LV-shHIF-1α-a	CCGGGTGATGAAAGAATTACCGAATCTCGAGATTCGGTAATTCTTTCATCACTTTTTG
LV-shHIF-1α-b	AATTCAAAAAGTGATGAAAGAATTACCGAATCTCGAGATTCGGTAATTCTTTCATCAC
LV-shCon-a	CCGGTTCTCCGAACGTGTCACGTTTCAAGAGAACGTGACACGTTCGGAGAATTTTTG
LV-shCon-b	AATTCAAAAATTCTCCGAACGTGTCACGTAAGTTCTCTACGTGACACGTTCGGAGAA
